# A case report of co-infection of Melioidosis and cutaneous Leishmaniasis

**DOI:** 10.1186/s12879-017-2639-7

**Published:** 2017-08-01

**Authors:** Isuru Chamika Indeewara Kahandawaarachchi, Gayani Samadara Premawansa, Wipula Warnasuriya, Malka Dassanayake, Enoka Corea

**Affiliations:** 1 North Colombo Teaching Hospital, Ragama, Sri Lanka; 2 Department of Microbiology, Faculty of Medicine, University of Colombo, Ragama, Sri Lanka

**Keywords:** Melioidosis, Cutaneous leishmaniasis, Co-infection

## Abstract

**Background:**

Leishmaniasis and melioidosis are frequently reported from the North Central Province of Sri Lanka. However, only one case of co-infection of the two diseases has been reported to date over the world. This is a case report of a patient who had co-infection with cutaneous leishmaniasis and melioidosis and was successfully treated and recovered from the illness.

**Case presentation:**

A 61 year old female patient with diabetes mellitus presented with fever for one month’s duration and was found to have hepatosplenomegaly and an ulcer over the left arm. She had elevated inflammatory markers and blood culture grew *Burkholderia pseudomallei* and serum was highly positive for melioidosis antibodies. A slit skin smear of the ulcer showed *Leishmania* amastigotes.

**Conclusion:**

Melioidosis and leishmaniasis are emerging infectious diseases in endemic countries and can be severe. The high prevalence rates in Sri Lanka should keep the treating physicians’ threshold for suspicion low for these two diseases.

## Background

Melioidosis and leishmaniasis are two important emerging infectious diseases in Sri Lanka [1–3]. Both these diseases are prevalent in the North Central Province.

Melioidosis, caused by *Burkholderia pseudomallei*, is thought to be transmitted via inoculation of contaminated soil and less commonly via aerosol inhalation, leading to a systemic infection which may be complicated by septicemia and/or disseminated abscess formation [4].

Leishmaniasis, caused by the *Leishmania* parasite occurs in the forms of cutaneous, muco-cutaneous, disseminated cutaneous and systemic infection. It is transmitted by the sand-fly [5].

An extensive literature search revealed only one case report on co-infection with melioidosis and cutaneous leishmaniasis [6].

Here we report a case of co-infection of melioidosis and cutaneous leishmaniasis in a patient residing at Padaviya in the North Central Province of Sri Lanka where both diseases are prevalent.

## Case presentation

A 61 year old female with a history of diabetes and hypertension, from Padaviya, Sri Lanka, presented to the Colombo North Teaching Hospital with a history of fever with chills and rigors of one month’s duration. She had developed an abscess over her left upper arm which ruptured into a non-healing ulcer coinciding with the onset of the fever.

**Fig. 1:**
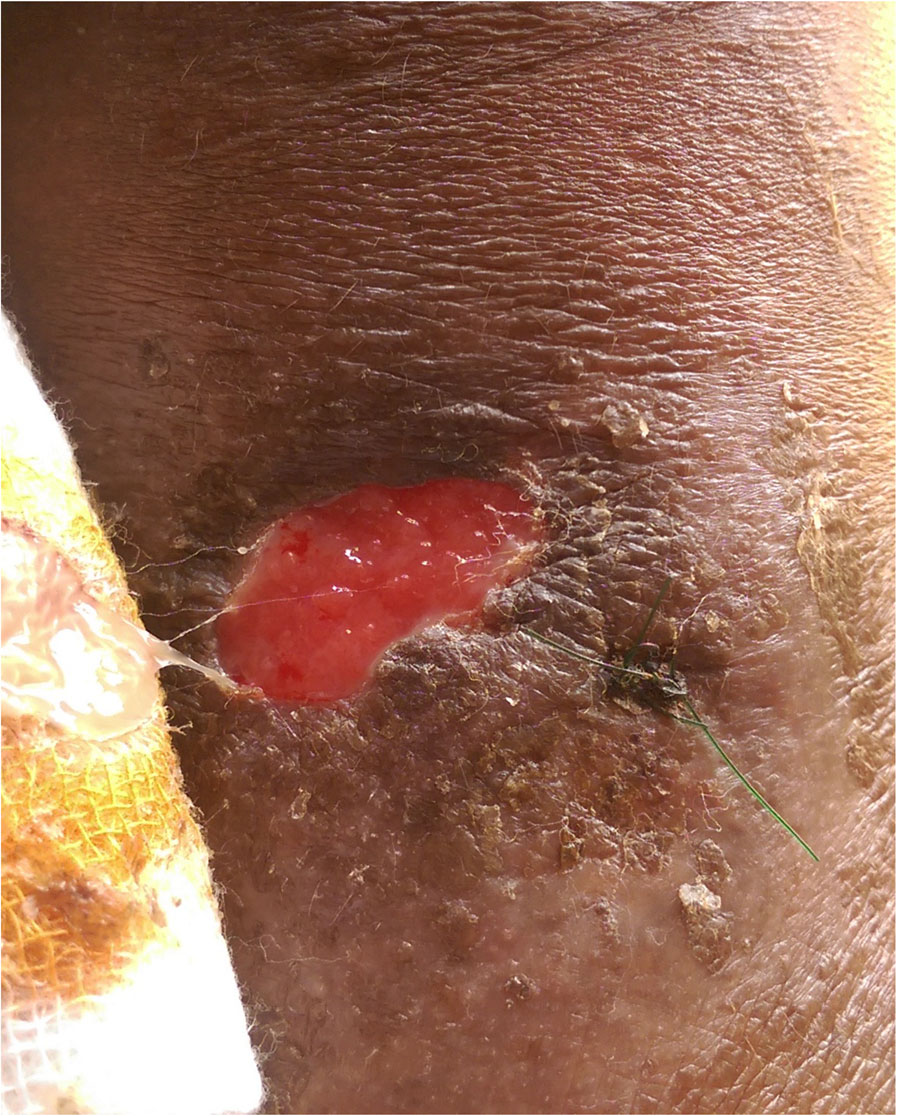
Ulcer over the left arm of the patient

On examination she was febrile and had an ulcer (Fig. 1) over her left upper arm which appeared unhealthy. Abdominal examination showed a smooth hepatomegaly 3 cm below the costal margin and a splenomegaly of 18 cm. Her respiratory, cardiovascular and neurological examinations were normal.

**Fig. 2:**
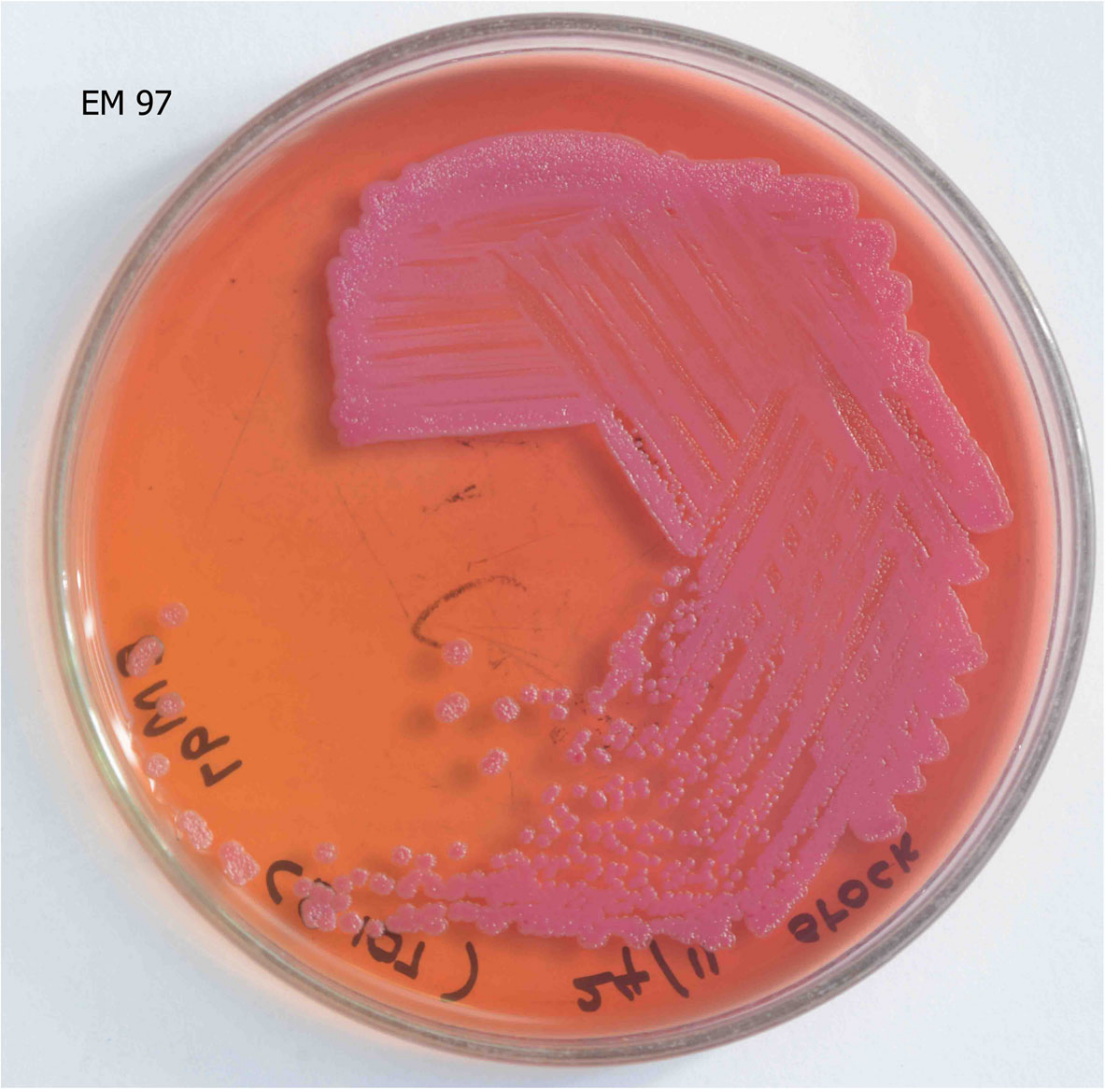
*B. pseudomallei* cultured in MacConkey agar

**Fig. 3:**
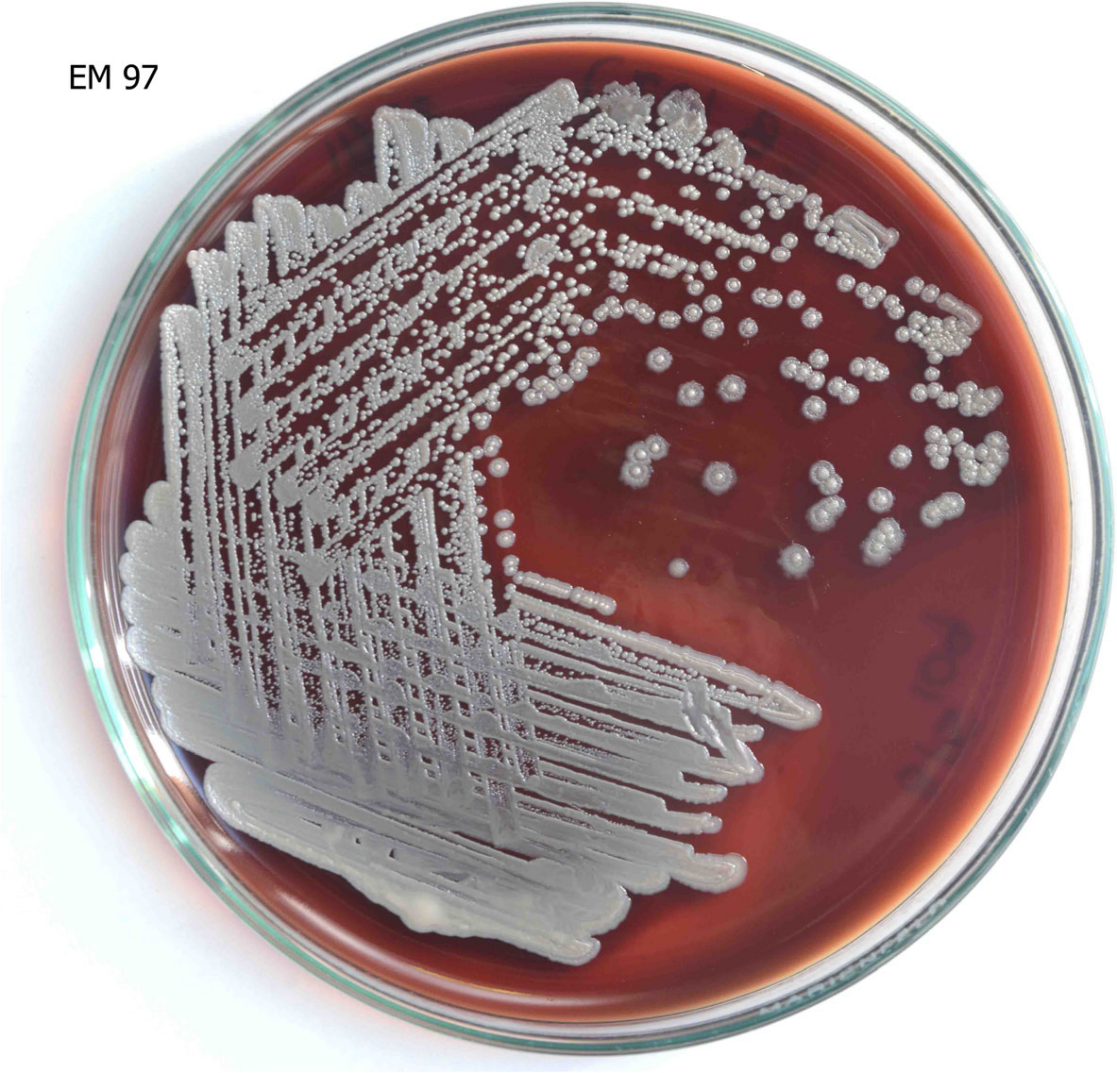
*B. pseudomallei* cultured in blood agar

Investigations revealed a neutrophilic leukocytosis with elevated inflammatory markers (C - reactive protein – 149 mg/L; erythrocyte sedimentation rate – 122 mm in first hour). The blood picture was in favor of an acute bacterial infection with neutrophils with toxic changes. Serum electrolytes, serum creatinine, and liver function tests were all normal. Her first two blood cultures did not grow any organisms but the third was positive for *B. pseudomallei* cultured in MacConkey (Fig. 2) and blood agar (Fig. 3) separately*.* The patient’s serum was positive for melioidosis antibodies by the indirect haemagglutination (IHA) test at a dilution of >1/10240 which was highly significant. The ultrasound scan and the contrast enhanced computerized tomography scan of the abdomen showed hepatosplenomegaly and mild pancreatitis but the serum amylase level was normal. There was no evidence of intra-abdominal abscesses. Fasting blood sugar level was 110 mg/dl and the capillary blood sugar levels during the hospital stay were well controlled with metformin 1 g 12 hourly and gliclazide 40 mg 12 hourly.

**Fig. 4:**
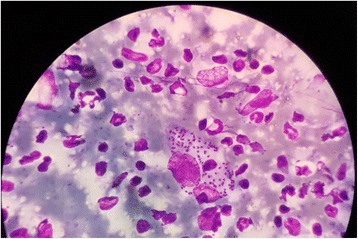
Slit skin smear of the patient showing *Leishmania* amastigotes

Since the ulcer remained non-healing, two slit skin smears were done, both of which showed *Leishmania* amastigotes (Fig. 4). However, serum antibodies against the Lc-rK39 antigen (using Kalazar Detect™ kit produced by Inbios International Inc.) of the organism was negative, excluding systemic infection. Wound swab for bacterial culture showed no growth.

**Fig. 5:**
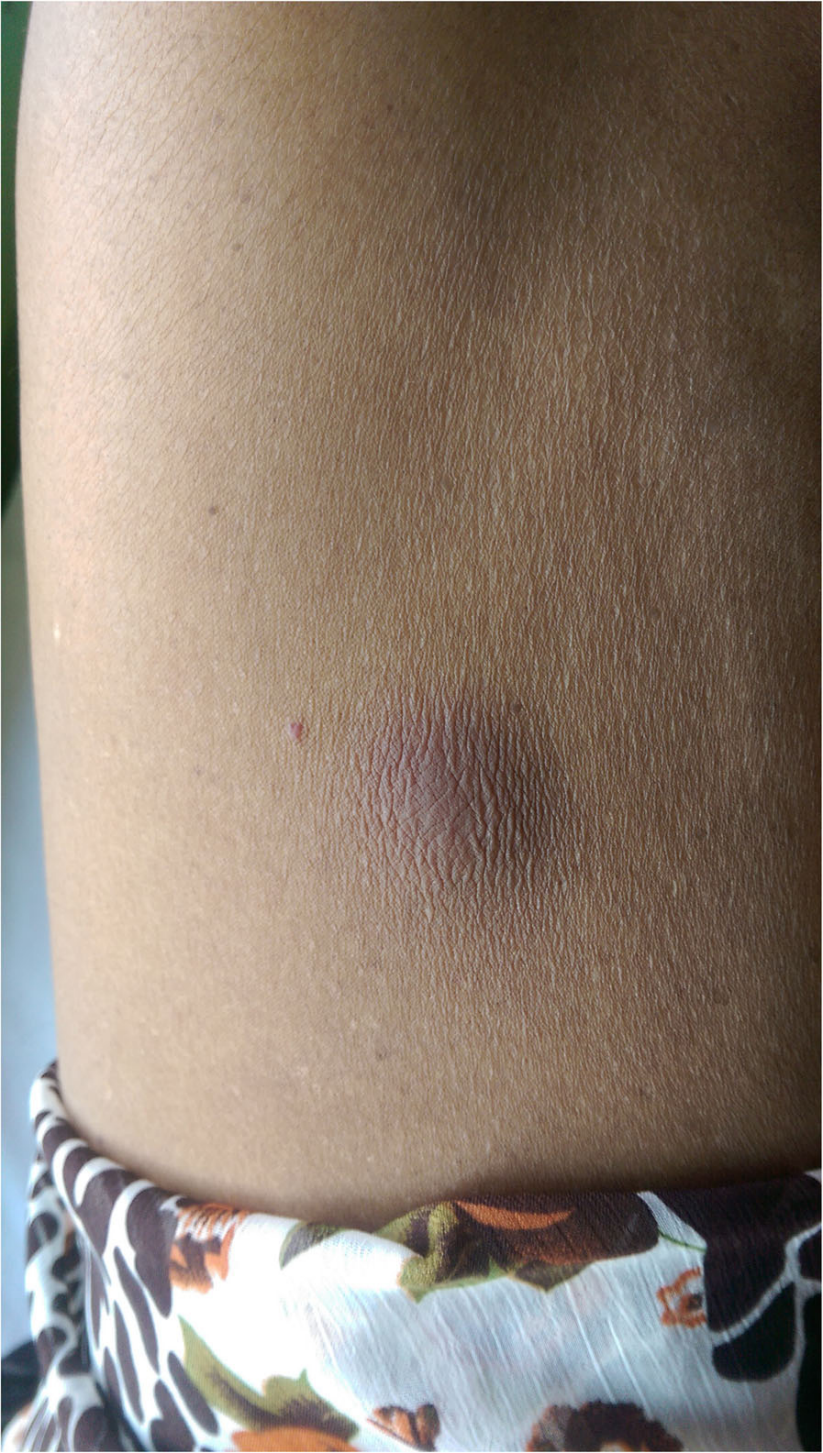
Abscess over the abdomen. Multiple abscesses such as these were present in the patient

The patient was initially commenced on intravenous ceftazidime 2 g six hourly but did not respond even after five days. She developed multiple abscesses all over the body (Fig. 5) one week into the hospital admission. However, pus cultures were negative for *B. pseudomallei.* She was then switched to a three week course of intravenous meropenem 1 g 8 hourly to which the fever responded. She was transferred to the Anuradhapura Teaching Hospital for continuation of meropenem for a further week and was subsequently discharged on eradication therapy with oral co-trimoxazole 960 mg (160 mg trimethoprim and 800 mg sulfamethoxazole) 12 hourly for six months and referred to the clinic for follow up.

The skin lesion was cauterized and no further treatment was administered since it was a local infection with the *Leishmania* parasite. The ulcer had healed almost completely by the time of discharge.

At 3 months’ follow up she remains afebrile, the ulcer healed completely and the hepatosplenomegaly absent.

## Discussion and conclusions

The pathogenesis of the two illnesses has been evaluated at molecular level and certain similarities have been found. It has been shown that increased TNF-α levels predispose to both diseases [7] and both illnesses are acted upon by a Type 1 T cell response which targets intracellular pathogens [8].

The ulcer on the arm of the patient probably occurred after a bite by the sand-fly which inoculated the leishmania organism in it. Secondary contamination of the ulcer with *B.pseudomallei* due to the high environmental prevalence of the organism could have caused the melioidosis infection. Diabetes mellitus would have predisposed the patient to acquiring the infection as well [9].

The presence of hepatosplenomegaly in the patient initially provoked the possibility of ‘kala azar’ or visceral leishmaniasis but since the patient was not very ill, was antibody negative and since she recovered with antibiotics to melioidosis, this assumption was discarded later [10, 11].

The diagnosis of melioidosis is often elusive but can be made through antibody assays and direct isolation via cultures [12]. The positive antibody titer and culture in this patient, together, pins the diagnosis of melioidosis.

Melioidosis treatment has two arms, the intensive phase and eradication phase. During the intensive phase, ceftazidime, meropenem or imipenem is used for a duration of 2–4 weeks. Co-trimoxazole may be added if the patient is poorly responding. The eradication phase uses co-trimoxazole or doxycycline up to a period of six months [13].

Cutaneous leishmaniasis can be treated with local cauterization or intra-lesional administration of sodium stibogluconate [14].

Since there was only one case report of a co-infection with both these illnesses and because there are set guidelines for the management of them individually, we treated the two infections separately.

As both melioidosis and leishmaniasis are emerging infectious diseases in Sri Lanka, physicians should have a high clinical suspicion when dealing with patients with pyrexia of unknown origin, especially when they’re from areas where the diseases are more prevalent.

## Abbreviations

IHA: Indirect haemagglutination
TNF-α: Tumor necrosis factor alpha
